# Rainbow trout CK9, a CCL25-like ancient chemokine that attracts and regulates B cells and macrophages, the main antigen presenting cells in fish

**DOI:** 10.18632/oncotarget.8163

**Published:** 2016-03-17

**Authors:** Carolina Aquilino, Aitor G. Granja, Rosario Castro, Tiehui Wang, Beatriz Abos, David Parra, Christopher J. Secombes, Carolina Tafalla

**Affiliations:** ^1^ Animal Health Research Center (CISA-INIA), Valdeolmos (Madrid), Spain; ^2^ Scottish Fish Immunology Research Centre, School of Biological Sciences, University of Aberdeen, Aberdeen, UK; ^3^ Animal Physiology Unit, Department of Cell Biology, Physiology and Immunology, School of Biosciences, Universitat Autonoma de Barcelona, Cerdanyola del Valles, Spain

**Keywords:** rainbow trout oncorhynchus mykiss, chemokines, CK9, CCL25, B cells, Immunology and Microbiology Section, Immune response, Immunity

## Abstract

CK9 is a rainbow trout (*Oncorhynchus mykiss*) CC chemokine phylogenetically related to mammalian CCL25. Although CK9 is known to be transcriptionally regulated in response to inflammation particularly in mucosal tissues, its functionality has never been revealed. In the current work, we have demonstrated that CK9 is chemoattractant for antigen presenting cells (APCs) expressing major histocompatibility complex class II (MHC II) on the cell surface. Among these APCs, CK9 has a strong chemotactic capacity for both B cells (IgM^+^ and IgT^+^) and macrophages. Along with its chemotactic capacities, CK9 modulated the MHC II turnover of B lymphocytes and up-regulated the phagocytic capacity of both IgM^+^ cells and macrophages. Although CK9 had no lymphoproliferative effects, it increased the survival of IgT^+^ lymphocytes. Furthermore, we have established that the chemoattractant capacity of CK9 is strongly increased after pre-incubation of leukocytes with a T-independent antigen, whereas B cell receptor (BCR) cross-linking strongly abrogated their capacity to migrate to CK9, indicating that CK9 preferentially attracts B cells at the steady state or under BCR-independent stimulation. These results point to CK9 being a key regulator of B lymphocyte trafficking in rainbow trout, able to modulate innate functions of teleost B lymphocytes and macrophages.

## INTRODUCTION

Chemokines or chemotactic cytokines are a family of cytokines that regulate immune cell migration under both inflammatory and normal physiological conditions. Constitutively expressed chemokines regulate homing, maturation and even microenvironmental segregation of immune cells within lymphoid organs, whereas, in response to inflammation, chemokines attract immune cells and modulate the activity of the recruited cells, thus conditioning the first steps of the immune response [1, 2]. Chemokines are defined by the presence of four conserved cysteine residues and are divided into four subfamilies based on the distinctive pattern of the two N terminal cysteines: CXC (α), CC (β), C and CX_3_C classes [3].

Chemokines are rapidly changing proteins and evolve more quickly than most other immune genes, a reflection of different infectious experiences of the host [4, 5]. In addition, the level of diversity of CC chemokines seems high in fish relative to mammals, making one-to-one orthologous relationships difficult to establish between mammalian and fish CC chemokines. In 2007, Peatman and Liu established seven large groups of fish CC chemokines through an extensive phylogenetic analysis using CC chemokine sequences from rainbow trout (*Oncorhynchus mykiss*), Atlantic salmon (*Salmo salar*), channel catfish (*Ictalurus punctatus*) and zebrafish (*Danio rerio*) along with mammalian CC chemokines [5]. These groups were named based on their mammalian membership as the CCL19/21/25 group, the CCL20 group, the CCL27/28 group, the CCL17/22 group, the macrophage inflammatory protein (MIP) group, the monocyte chemotactic protein (MCP) group and a fish-specific group. Among these, the CCL19/21/25 group is one of three groups showing higher evolutionary conservation between teleost fish and mammalian CC chemokines; however, the authors observed that most fish genes in this group were most closely related to CCL25, with lower similarity to CCL21. The branching pattern indicated that ancient vertebrates had multiple CCL25/21-like genes before the diversification of teleost fish species, however, recent phylogenetic analyses suggest that teleost fish may lack true CCL21 orthologues [6]. In some fish species, such as zebrafish, two genes that seem true orthologues of CCL25 are present [7]. On the other hand, the CCL19 subclade in the CCL19/21/25 group is particularly well established and constitutes an independent gene [5].

In rainbow trout, after the designation of the first CC chemokine identified as CK1 [8] this nomenclature was maintained for subsequently discovered CC chemokine genes, bringing up the total number of CC chemokines to eighteen [9, 10]. Among them, CK9 is a CC chemokine ascribed within the CCL19/21/25 group, closely related to mammalian and zebrafish CCL25 [5]. This designation was later confirmed in an independent phylogenetic analysis [11]. Thus far, although multiple studies have been undertaken addressing the transcriptional regulation of CK9 in response to diverse stimuli and throughout different tissues, pointing to a preferential mucosal role [12-14], its biological activity has never been determined.

In mammals, CCL25 signals exclusively through CCR9 [15] which is mainly expressed in the thymus where it regulates the trafficking of developing T cells [16] and in the intestine where it recruits T cells to Peyer's patches [15, 17]. Interestingly, CCL25 is also chemotactic for IgA antibody secreting cells (ASCs), while IgM or IgG ASCs respond poorly [18, 19]. Similarly, Toll like receptor 2 (TLR2) ligands have been shown to induce IgA and CCR9 expression in circulating B lymphocytes [20], and during the course of an acute rotavirus infection both IgA and IgM ASCs express CCR9 and are able to migrate to CCL25 [21]. Likewise, mucosal immunization against *Salmonella* provokes a robust migration of specific IgA- and IgM-ASCs towards CCL25 [22]. CCL25 also has chemotactic potential towards activated macrophages [23] and CCR9^+^ macrophages are known to play a prominent role in acute liver inflammation [24] and rheumatoid arthritis [25]. Interestingly, it has recently been shown that only human M1 macrophages (classically activated macrophages) express CCR9 and migrate towards CCL25, while M2 macrophages (alternatively activated macrophages) do not have this ability [26].

In teleost fish, different B lymphocyte subsets can be found that are defined by their expression of the three immunoglobulin (Ig) isotypes present in fish. IgD^+^IgM^+^ B cells are present in all teleost species analyzed thus far and represent the majority of B lymphocytes in fish [27]. IgD^+^IgM^−^ B cells have been reported in channel catfish [28] and in rainbow trout gills [29], but their role in immunity has not been clarified yet. Finally, a lineage of B cells uniquely expressing IgT/Z has been reported in some species [30, 31], where they seem particularly important for mucosal responses [31, 32].

In the current work, we have studied the biological activity of rainbow trout CK9, characterizing the specific cell types that are attracted to this chemokine, and then determined the bioactivity of CK9 on the recruited cells. Our results show that CK9 is a chemoattractant for antigen presenting cells (APCs), including B lymphocytes (both IgM^+^ and IgT^+^ B cells) as well as macrophages. CK9 regulated the phagocytic capacity of both macrophages and IgM^+^ cells, and increased the major histocompatibility complex class II (MHC II) molecule turnover in both B lymphocyte subsets. Unlike other mammalian chemokines, CK9 did not show lymphoproliferative effects, but specifically increased the survival of IgT^+^ lymphocytes. Interestingly, the chemoattractant capacity of CK9 was significantly increased when leukocytes were pre-incubated with a T-independent antigen such as TNP-LPS but to a lesser extent when a T-dependent antigen was used. On the other hand, B cell receptor (BCR) cross-linking drastically reduced the capacity of B lymphocytes, especially IgM^+^ cells, to migrate to CK9. Our results suggest that CK9 is an ancient chemokine that regulates the innate functions of teleost B lymphocytes and macrophages, and suggests that rainbow trout CK9 and its homologues in other fish species are key modulators of B lymphocyte trafficking in teleost fish.

## RESULTS

### CK9 attracts and activates RTS11 rainbow trout macrophages

Recombinant CK9 was produced in order to study its bioactivity. A protein of the expected size of 9.61 kDa was induced by IPTG stimulation of transformed BL21 cells, purified under denaturing conditions, refolded *in vitro* and re-purified under native conditions. The recombinant CK9, when added to RTS11 cells at up to 1000 ng/ml, had no effects on the expression of interleukin 1β (IL-1β) and tumor necrosis factor α (TNF-α), which are known to be up-regulated by liposaccharide (LPS) in this system [33, 34], confirming that LPS contamination in the recombinant preparations was negligible [35].

**Figure 1:**
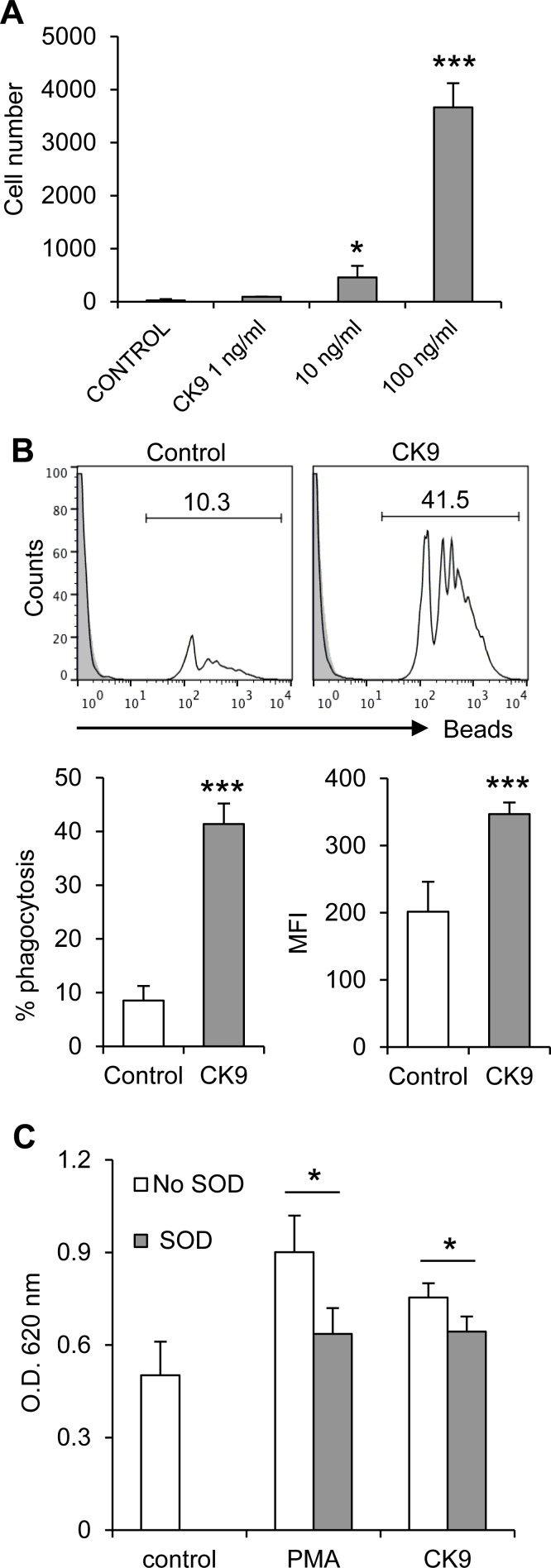
Effect of CK9 on rainbow trout RTS11 macrophages **A.** Chemotaxis assay in which different CK9 doses were introduced in the bottom wells of transwell chambers, whereas RTS11 cells were dispensed into the upper wells. After 2 h of incubation at 20°C, the number of cells that had migrated to the bottom of the wells was quantified by flow cytometry. Average numbers of migrating cells are shown. **B.** RTS11 cells were incubated with Crimson Red fluorescent beads at a ratio of 1:10 (cell:beads) for 3 h in the presence or absence of CK9 (100 ng/ml). The cells were then analyzed by flow cytometry to measure the fluorescence of internalized beads, as shown in the representative histograms. Solid grey overlays represent controls with no beads. Percentages of cells containing beads were determined and average percentage of phagocytic cells was calculated, as well as median fluorescence intensity (MFI) of the internalized beads. **C.** RTS11 cells were incubated with NBT (1 mg/ml) in combination with either PMA (1 μg/ml) or CK9 (100 ng/ml). SOD (300 units/ml) was included in some wells to verify the specificity of the reaction. After 30 min of incubation at 20°C, NBT reduction was measured as the optical density at 620 nm. Data are shown as average readings (*n* = 6 assays, mean + SD). Statistical analysis was performed in each case, where * means *p* ≤ 0.05 and *** means *p* ≤ 0.005.

The chemotactic activity of recombinant CK9 was first tested on the rainbow trout macrophage cell line RTS11. Using transwell migration chamber assays, we analyzed the effect of different doses of CK9 on the migratory capacity of RTS11 macrophages towards this chemokine and observed that CK9 attracted unstimulated trout macrophages in a dose-dependent manner, reaching very high significant levels of chemotaxis at 100 ng/ml CK9 (Figure 1A). When CK10, another chemokine produced in parallel under the same conditions was tested using the same doses, no RTS11 cell migration was ever observed. Since chemokines not only recruit immune cells to sites of inflammation, but also have the capacity to activate the recruited cells [36], we investigated whether CK9 had an impact on the phagocytic response of RTS11 macrophages. After incubation with 1 μm polystyrene-based fluorescent beads for 3 h, RTS11 macrophages showed a modest phagocytic capacity (an average of 9% of cells), which was dramatically increased by the presence of CK9 during the incubation, leading to an average of 41% of cells being phagocytic (Figure 1B). CK9 not only increased the number of phagocytic cells but also their capability to internalize beads, since the median fluorescence intensity (MFI) increased from 201.6 (control) to 346.8 (CK9) (Figure 1B, bar plots). A hallmark of activated phagocytes is the generation of reactive oxygen species during the phagocytosis-associated respiratory burst [37], so we also analyzed the impact of CK9 on the respiratory burst activity of RTS11 cells. Interestingly, CK9 significantly induced respiratory burst activity in rainbow trout macrophages, to levels almost comparable to those obtained when RTS11 macrophages where incubated with the inducer PMA (Figure 1C). Furthermore, SOD significantly reduced the respiratory burst induced by either PMA or CK9, indicating specificity for both. Altogether, these data indicate that CK9 attracts trout macrophages and activates their phagocytic and microbicidal abilities.

### CK9 attracts B cells from spleen, head kidney and blood as well as myeloid cells from head kidney

**Figure 2:**
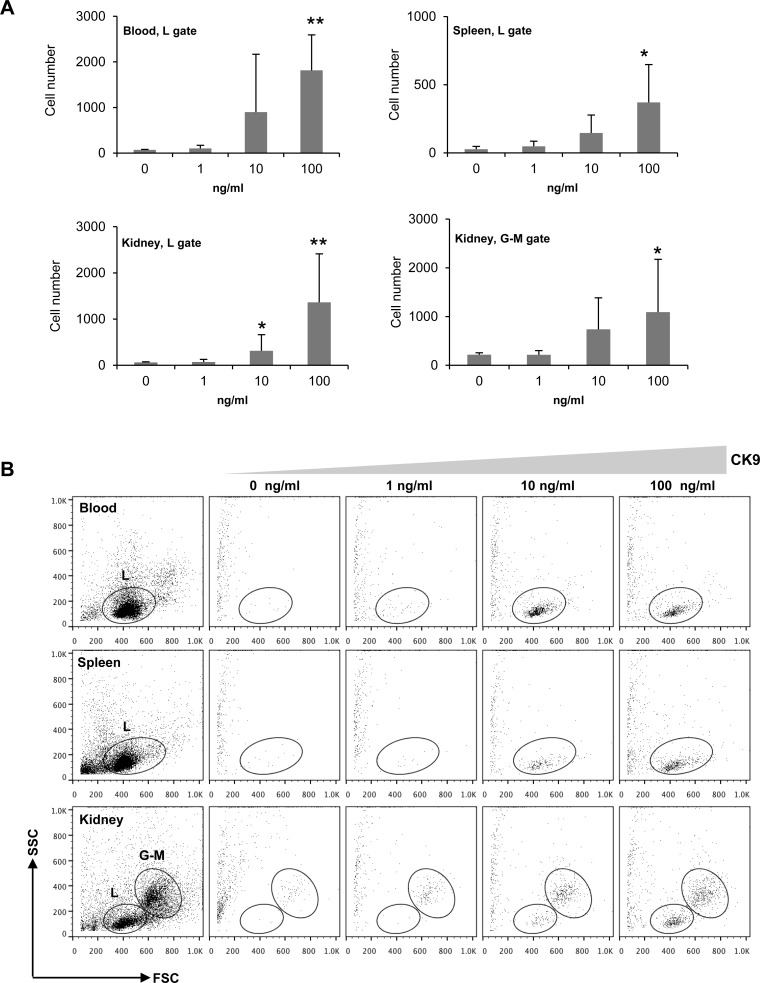
Chemotactic activity of CK9 on rainbow trout leukocyte populations Different doses of CK9 were introduced into the bottom well of a transwell chamber, whereas leukocytes from blood, head kidney or spleen were dispensed into the upper wells. After 2 h of incubation at 20°C, the number of cells that had migrated towards CK9 was quantified by flow cytometry. **A.** Average numbers of migrating cells (*n* = 6 fish, mean ± SD). Statistical analysis was performed in each case, where * means *p* ≤ 0.05 and ** means *p* ≤ 0.01. **B.** Representative dot plots. L: Lymphoid gate, G-M: Granulocyte-Macrophage gate.

We next characterized the chemotactic activity of CK9 on rainbow trout unstimulated leukocytes from spleen, head kidney and peripheral blood (PBLs) using different CK9 doses. CK9 was a chemoattractant for trout leukocytes from spleen, head kidney and peripheral blood (Figure 2). The effect was dose-dependent, reaching significant levels of chemotaxis already with 10 ng/ml in the case of kidney, and with 100 ng/ml of CK9 in all tissues analyzed (Figure 2B). Among the leukocytes attracted, a lymphocyte-like population (based on its SSC/FSC profile) was present in all tissues (Figure 2A, L population), whereas, interestingly, a migrated population of larger cells on the granulocyte-macrophage gate was also visible in head kidney leukocyte cultures (Figure 2A, G-M population), an organ where these cell types are abundant. When CK10, another chemokine produced in parallel under the same conditions was tested using the same doses, no cell migration was ever observed in any of the tissues tested.

Chemokines signal through G-coupled chemokine receptors which are specifically blocked by pertussis toxin (PTX), thus we also studied whether CK9-induced migration could be blocked by the pre-incubation of leukocytes with PTX. We found that pre-incubation of splenocytes with PTX prior to the chemotaxis assay had no effect on leukocyte viability but significantly blocked the migration to CK9 in a dose-dependent fashion (Figure S1), demonstrating that CK9 signals through a G-coupled receptor.

**Figure 3:**
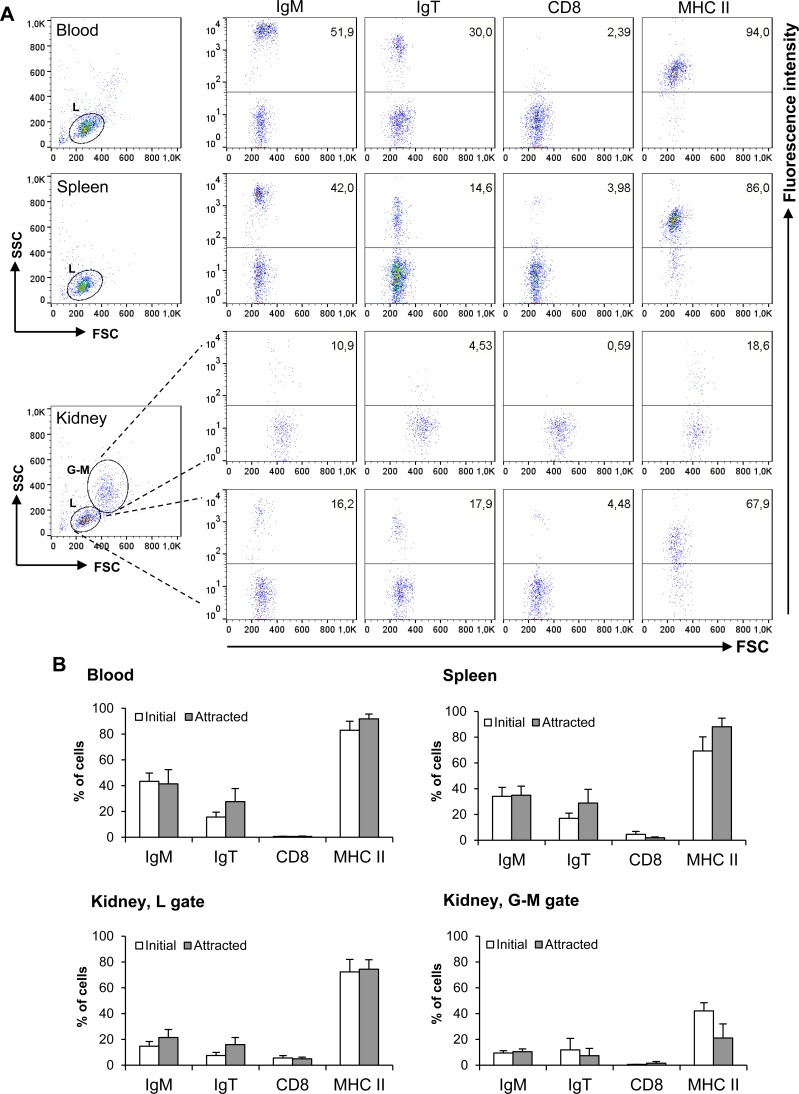
Characterization of leukocyte populations attracted to CK9 A single dose of 100 ng/ml was introduced into the bottom well of a transwell chamber, whereas leukocyte populations from blood, head kidney or spleen were dispensed into the upper wells. After 2 h of incubation at 20°C, the cells that had migrated to the bottom of the wells were harvested and stained with anti-IgM, anti-IgT, anti-CD8α or anti-MHC II specific antibodies. **A.** Representative dot plots and staining patterns for each tissue are shown. **B.** Mean percentage of specific leukocyte populations in initial cultures and in CK9-attracted cells (*n* = 5).

Subsequently, to characterize the nature of the attracted cells, we performed a chemotaxis assay using a single CK9 dose (100 ng/ml), and then incubated the migrated cells with specific antibodies against IgM, IgT, CD8α and MHC II. Within the lymphoid gate, the chemokine mainly attracted MHC II^+^ B cells in leukocyte cultures from either spleen, head kidney or blood, accounting for an average of 74-92% of the migrated cells within the lymphoid gate (Figure 3), revealing that CK9 is a strong chemoattractant for APCs. Surprisingly, only an average of 42% of the cells attracted to CK9 in the kidney that fall within the myeloid gate are MHC-II^+^ (Figure 3), thus the nature of the remaining cell types attracted remains unknown given the current availability of antibodies against trout leukocyte populations. Since APCs include macrophages, dendritic cells (DCs) and B cells, we also stained the migrated cells with anti-IgM and anti-IgT to study if B cells were attracted by CK9. Both IgM^+^ and IgT^+^ cells were among the main attracted cell types in spleen and blood, since together they accounted for an average of 64% and 69% of the attracted cells respectively (Figure 3). In contrast, the percentage of IgM^+^ and IgT^+^ cells in the attracted population from kidney was lower, suggesting that in this organ, other APCs different than B cells are also strongly attracted towards CK9. Finally, the levels of CD8α^+^ cells detected in the migrated cells was quite low (Figure 3), however, it is true that the percentage of CD8α^+^ cells was also quite low in leukocyte suspensions from these tissues, as previously reported [38]. Thus, our results reveal that CK9 is a common chemoattractant for different types of APCs, which in the case of B cell-rich tissues such as spleen or blood, mainly account for IgM^+^ and IgT^+^ lymphocytes.

### CK9 increases the phagocytic activity of naïve IgM^+^ B cells

**Figure 4:**
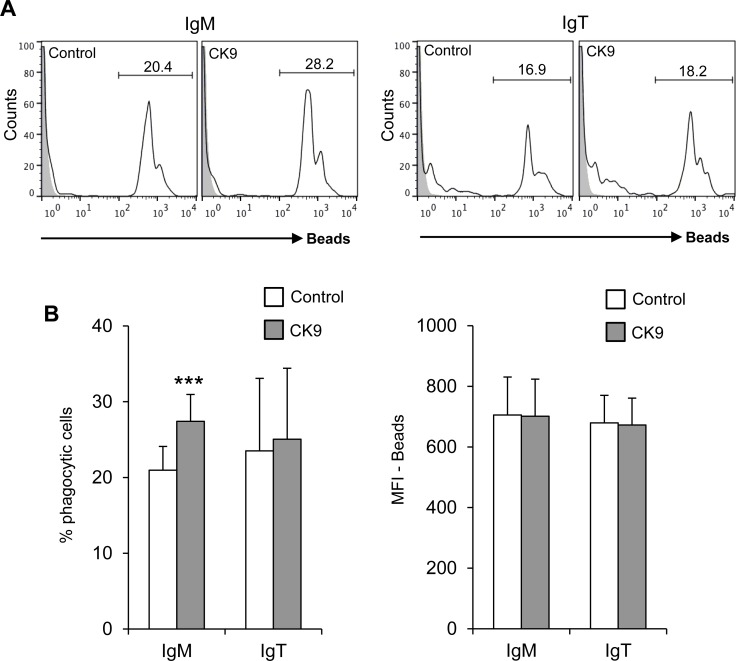
Effect of CK9 on the phagocytic capacity of B lymphocytes PBLs were incubated with Crimson Red fluorescent beads at a ratio of 1:10 (cell:beads) for 3 h in the presence or absence of CK9 (100 ng/ml). **A.** Cells were stained with either anti-IgM or anti-IgT antibodies and then analyzed by flow cytometry, as shown in the representative histogram. **B.** Average percentages of phagocytic cells were also calculated (left bar plot) as well as median fluorescence intensities (MFI) (right bar plot) of the beads in order to determine the phagocytic capacity and the effects of CK9. Data are shown as mean ± SD (*n* = 7 fish). Statistical analysis was performed in each case, where *** means *p* ≤ 0.005.

Since rainbow trout IgM^+^ and IgT^+^ B cells have a potent phagocytic activity [31, 39], we wanted to determine if CK9 could also modulate this activity, as in the case of RTS11 cells. To carry out this experiment, PBLs were incubated with 1 μm polystyrene-based fluorescent beads for 3 h in the presence or absence of 100 ng/ml CK9. Cells were then labelled with anti-IgM and anti-IgT mAbs, to determine the phagocytic capacity of each specific subpopulation. CK9 increased the number of phagocytic IgM^+^ B cells with very high statistical significance (Figure 4A), but had no effect on the number of phagocytic IgT^+^ B cells (Figure 4). As expected, CK9 did not increase the MFI of IgT^+^ B cells, but surprisingly, also had no effect on IgM^+^ B cells (Figure 4B). Thus, it seems that CK9 is able to increase the number of IgM^+^ B cells that can phagocytose an average number of beads, but is not able to increase the capability of IgM^+^ B cells to ingest a higher bead number.

### CK9 activates the internalization of cell surface MHC II in resting B cells

**Figure 5:**
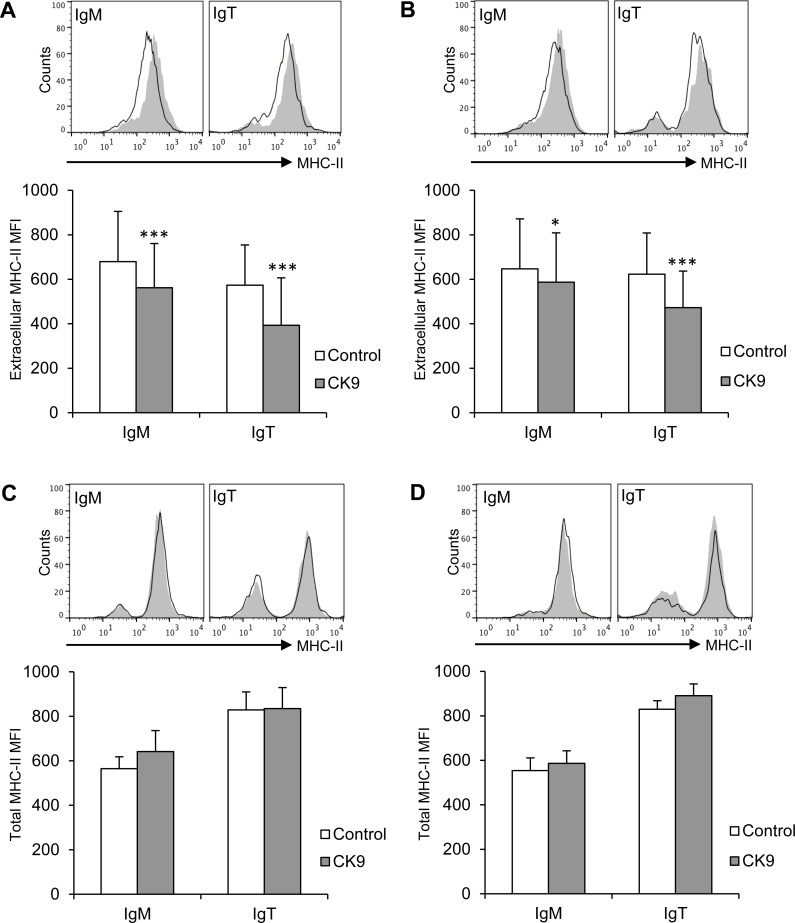
Effect of CK9 on the expression of MHC II on trout B lymphocytes Spleen and blood leukocytes were incubated with CK9 (100 ng/ml) or control media for 24 h at 20°C. After that time, MHC II cell surface expression on blood **A.** and spleen **B.**. IgM^+^ or IgT^+^ B cells was estimated by flow cytometry using a specific anti-MHC-II monoclonal antibody in combination with an anti-IgM or an anti-IgT antibody. In parallel, total MHC-II levels (intracellular plus extracellular) on blood **C.** and spleen **D.** B cells was estimated by flow cytometry using a specific anti-MHC II monoclonal antibody after fixation and permeabilization of previously stained IgM^+^ and IgT^+^ cells. Plots show MHC II expression in IgM^+^ or IgT^+^ cells from CK9-treated cultures (line) compared to MHC II expression in corresponding control cultures (grey plot, overlaid). Isotype control is represented by a dashed line. MHC II mean fluorescence intensity (MFI) was calculated for each sample and plotted. Data are shown as MFI ± SD (*n* = 6 fish). Statistical analysis was performed in each case, where * means *p* ≤ 0.05 and *** means *p* ≤ 0.005.

Since CK9 was able to enhance the phagocytosis of extracellular particles by IgM^+^ cells, we additionally tested whether this chemokine influenced MHC II trafficking on both IgM^+^ and IgT^+^ B lymphocyte populations. After a 24 h exposure to CK9, both IgM^+^ and IgT^+^ peripheral blood B cells significantly downregulated their levels of surface MHC II (Figure 5A). A similar result was observed when splenic B cells were analyzed, since CK9 induced a very significant decrease of surface MHC II on IgT^+^ B cells, and, to a lesser extent, on IgM^+^ B cells (Figure 5B). Interestingly, total levels (intracellular plus extracellular) of MHC II were not affected by CK9 on either B cell population from blood (Figure 5C) or spleen (Figure 5D). The fact that surface MHC II levels decrease on B cells while total levels remains the same demonstrates that CK9 activates MHC II trafficking from the membrane to intracellular compartments.

### CK9 has no proliferative effects but increases the survival of IgT^+^ cells

**Figure 6:**
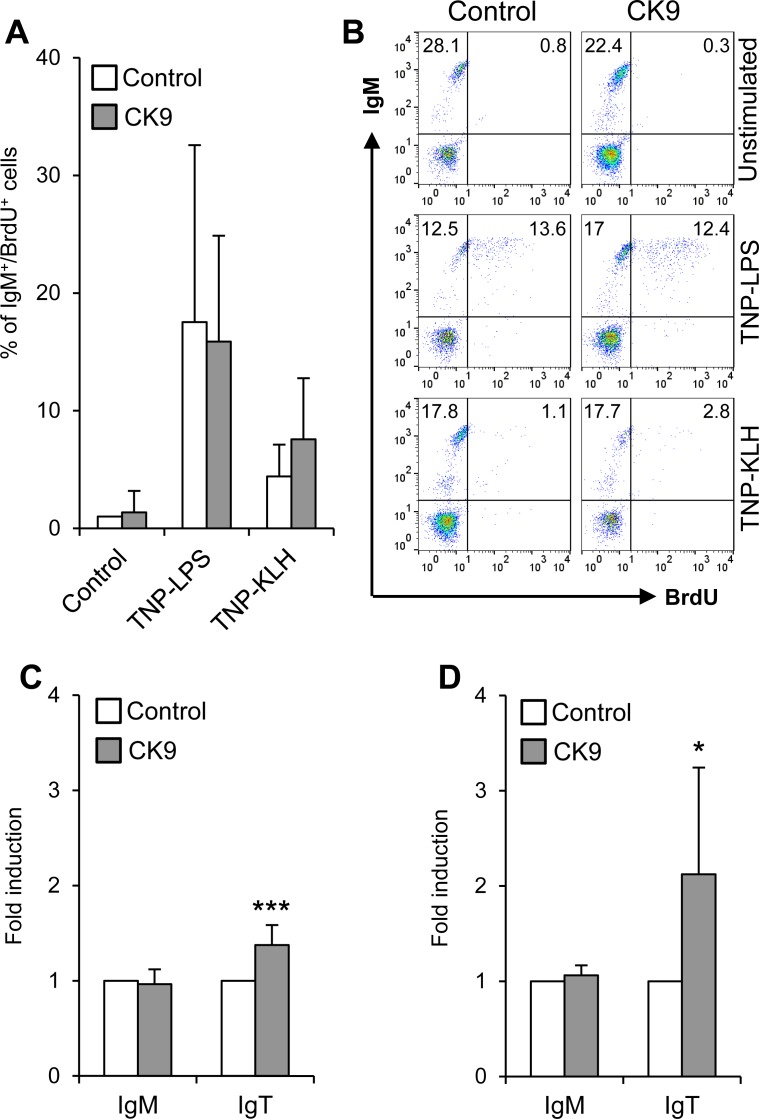
Effect of CK9 on IgM^+^ and IgT^+^ B cell survival and proliferation **A.** PBLs were incubated with TNP-LPS or TNP-KLH (5 mg/ml) in the absence or presence of CK9 (100 ng/ml) for 3 days at 20°C. Controls without stimuli, with or without CK9, were also included. Bromodeoxyuridine (BrdU, 10 μM) was then added to the cultures and cells were incubated for an additional 24 h. After that time, trout cells were collected and stained with anti-IgM, then fixed, permeabilized and stained with an anti-BrdU antibody and analysed by flow cytometry. The histogram shows the percentage of proliferating IgM^+^ cells (BrdU^+^ / IgM^+^). Data are shown as mean % ± SD (*n* = 5 fish). **B.** Representative flow cytometry charts are also shown. Survival of PBLs **C.** and splenocytes **D.** incubated with control medium or medium containing CK9 (100 ng/ml) was also determined after 4 days of culture at 20°C. After that time, cultures were stained with anti-IgM and anti-IgT antibodies to quantify survival of IgM^+^ and IgT^+^ B cells in the cultures. The number of cells in each culture was normalized to each control value, thus representing the effect of CK9 on survival as fold induction. Data are shown as mean ± SD (*n* = 5 fish). Statistical analysis was performed in each case, where * means *p* ≤ 0.05 and *** means *p* ≤ 0.005.

Unlike other mammalian chemokines with lymphoproliferative effects on B cells [40], CK9 did not induce on its own the proliferation of IgM^+^ B cells (Figure 6A, B) or IgT^+^ cells (data not shown). When blood B cells were exposed to TNP-LPS as a T-independent antigen and TNP-KLH as a T-dependent antigen [41], we observed that TNP-LPS was much more efficient in triggering a strong proliferative response of naïve IgM^+^ B lymphocytes than TNP-KLH (Figure 6A, 6B). However, the combination of CK9 together with TNP-LPS or TNP-KLH produced no significant modulation of the proliferative response to either of the two stimuli (Figure 6A, 6B). Surprisingly, neither TNP-LPS nor TNP-KLH were capable of inducing a proliferative response of blood or spleen IgT^+^ B lymphocytes (data not shown).

Despite having no lymphoproliferative effects, CK9 significantly increased the survival of IgT^+^ B cells in both PBLs (Figure 6C) and splenocytes (Figure 6D) after 4 days of culture. This effect is highly specific for this population, as it was not visualized for IgM^+^ cells. Given the transcriptional profile of CK9, these results strongly suggest that CK9 contributes to the maintenance of IgT^+^ B cell viability in the mucosal surfaces.

### The pre-stimulation of leukocytes modulates their migratory capacities towards CK9

**Figure 7:**
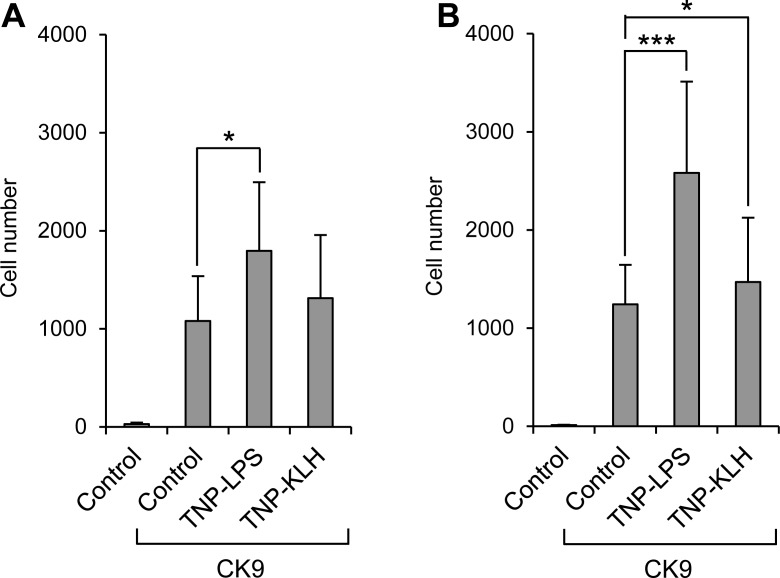
Effect of B lymphocyte stimulation on the migratory capacity of leukocytes towards CK9 Leukocyte populations from blood **A.** or spleen **B.** were incubated with TNP-LPS or TNP-KLH (5 mg/ml) or control medium, for 16 h at 20°C. After that time, the cells were introduced into the upper wells of a transwell chamber, whereas 100 ng/ml CK9 were introduced into the bottom well of the chambers. After 2 h of incubation at 20°C, the number of cells that had migrated to the bottom of the wells was quantified by flow cytometry. Data are shown as mean ± SD (*n* = 5 fish). Statistical analysis was performed in each case, where * means *p* ≤ 0.05 and *** means *p* ≤ 0.005.

We also studied whether the pre-stimulation of B lymphocytes altered their capacity to migrate towards CK9 using TNP-LPS and TNP-KLH antigens. In blood, only TNP-LPS significantly increased the capacity of leukocytes to migrate to CK9 (Figure 7A). In spleen, although both TNP-LPS and TNP-KLH gave increased numbers of migrated cells (Figure 7B), the effects of TNP-LPS were much stronger than those of TNP-KLH. Since TNP-LPS has strong lymphoproliferative effects, it was possible that the increased chemotaxis obtained in TNP-LPS-stimulated cultures was a consequence of an increased number of IgM^+^ cells. Thus, to rule out this confounding factor, we repeated this experiment, adjusting the number of cells not only prior to the 24 h stimulation with TNP-LPS, but again before the cells were introduced into the chemotaxis chambers. In these conditions, TNP-LPS-stimulated cells again showed a significantly increased migration towards CK9 with similar percentages of IgM^+^ and IgT^+^ cells (data not shown), supporting the statement that pre-stimulation of B lymphocytes by a T-independent antigen increases their responsiveness to CK9.

### BCR engagement in IgM^+^ cells inhibits their capacity to migrate towards CK9

**Figure 8:**
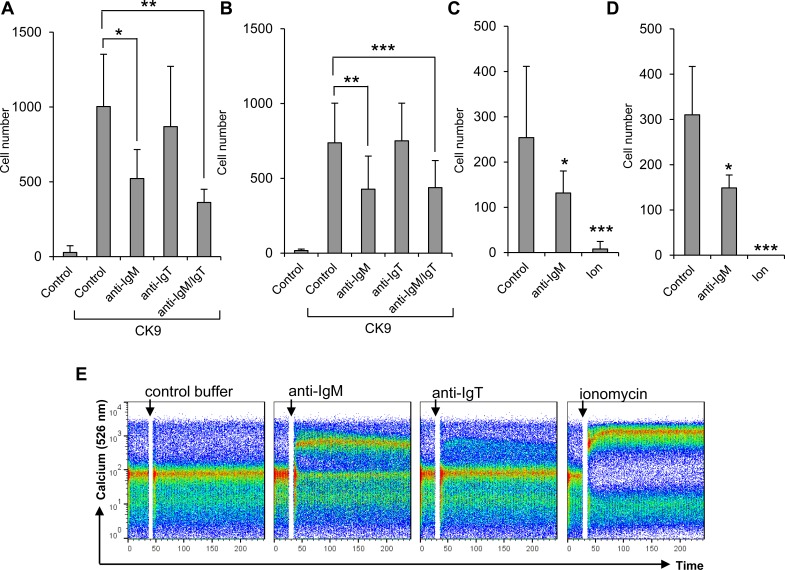
Effect of BCR cross-linking on the migratory capacity of leukocytes towards CK9 Leukocyte populations from blood **A.** or spleen **B.** were incubated with 0.5 mg/ml of anti-IgM antibody, 0.5 mg/ml of anti-IgT antibody or a combination of both, for 24 h at 14°C. After that time, the cells were introduced into the upper wells of a transwell chamber, whereas 100 ng/ml CK9 were introduced into the bottom well of the chambers. After 2 h of incubation at 20°C, the number of cells that had migrated to the bottom of the wells was quantified by flow cytometry. In parallel, leukocyte populations from blood **C.** or spleen **D.** were incubated with 0.5 mg/ml of anti-IgM or 1 mM ionomycin for 5 min at 14°C. After that time, the cells were introduced into the upper wells of a transwell chamber, whereas 100 ng/ml CK9 were introduced into the bottom well of the chambers. After 2 h of incubation at 20°C, the number of cells that had migrated to the bottom of the wells was quantified by flow cytometry. Average numbers of migrating cells (*n* = 5-7 fish, mean ± SD) are shown. Statistical analysis was performed in each case, where * means *p* ≤ 0.05, ** means *p* ≤ 0.01 and *** means *p* ≤ 0.005. **E.** PBLs were loaded with Fluo-3 AM to follow the amount of intracellular calcium upon stimulation with 0.5 mg/ml of anti-IgM, 0.5 mg/ml of anti-IgT or 1mM ionomycin (positive control) by flow cytometry. Data are representative of five independent experiments.

Contrary to the effects obtained by stimulation with a T-independent antigen, we observed that BCR cross-linking with an anti-IgM antibody significantly suppressed the capacity of IgM^+^ cells to migrate towards CK9 both in blood (Figure 8A) and spleen (Figure 8B). BCR cross-linking through anti-IgM is known to mimic a high affinity antigen, whereas TNP-LPS signals through low affinity membrane Igs and pattern recognition receptors which induce a much weaker stimulatory signal on B cells [42]. The effect of anti-IgM was visible when cells were incubated with the antibody for 24 h (Figure 8A, 8B) or even 5 min (Figure 8C, 8D), despite the fact that incubation with this antibody for these time periods had no effect on the cell viability of the cultures as determined by propidium iodide (data not shown). This IgM-mediated suppression was observed when the antibody was added alone or in combination with the anti-IgT antibody but not when the anti-IgT antibody was used on its own (Figure 8A, 8B). The reason for this lack of effect in response to the anti-IgT antibody could be a result of lower percentages of IgT^+^ cells in the leukocyte cultures, or might be a consequence of a lower activating capacity for this antibody. Either way, the anti-IgT antibody provokes a weak calcium influx in blood leukocytes whereas the anti-IgM provoked a much stronger calcium influx (Figure 8E) that confirms BCR cross-linking and a strong B lymphocyte activation [43]. To unequivocally confirm that the inhibition of responsiveness to CK9 is due to B cell activation with the consequent calcium influx, we also incubated blood and spleen leukocytes with ionomycin for 5 min to provoke maximum intracellular calcium release. In this case, there was a complete blockage of the migratory capacities of leukocytes towards CK9 (Figure 8C, 8D), confirming that when B lymphocytes are fully activated by a high affinity antigen (such as that mimicked by anti-IgM) they no longer migrate towards CK9.

## DISCUSSION

In the current study, we have established the chemoattractant potential of CK9, an ancient chemokine with close homology to mammalian CCL25, towards trout MHC II^+^ APCs, including B lymphocytes (both IgM^+^ and IgT^+^ B cells) and macrophages, and established its potential to regulate specific immune functions in the different recruited cell types.

If we compare the CK9-attracted cell types to those recruited by CCL25 in mammals, significant differences are observed. While CK9 attracts unstimulated RTS11 macrophages and kidney MHC II^+^ myeloid cells, CCL25 has only been shown to attract previously activated macrophages [23-25]. In humans, CCL25 has also been shown to promote the differentiation of monocytes to macrophages [25] and likewise, we have observed an activation of macrophages in response to CK9, specifically on their phagocytic and respiratory burst activity, which should increase their antigen presenting and microbicidal potential.

Concerning B lymphocytes, although CCL25 exclusively attracts B lymphocytes that have differentiated towards ASCs [18-22], CK9 is a strong chemoattractant for naïve B lymphocytes in trout. Interestingly, B lymphocytes in teleost fish are known to produce large amounts of natural antibodies in physiological conditions [44] and are known to share many functions with mammalian B1 lymphocytes responsible for the secretion of natural antibodies in homeostasis [45], such as for example their phagocytic capacity [46, 47]. On the other hand, when the leukocyte cultures were stimulated with a T-independent antigen such as TNP-LPS, known to induce antibody secretion (data not shown), the capacity of B lymphocytes to migrate towards CK9 was significantly increased. This effect was only observed to a certain extent when a T-dependent antigen such as TNP-KLH was used, thus demonstrating that resting B lymphocytes are best activated for CK9 responsiveness under T-independent stimulation. Similarly, trout B lymphocytes strongly proliferated in response to TNP-LPS but not to TNP-KLH. All these results, suggest a great amount of functional similarities between mammalian B1 cells and teleost B lymphocytes and in consequence, it would be of interest to study if CCL25 is chemotactic for mammalian B1 subpopulations as it is for ASCs.

Overall, our results point to a key role of this chemokine in the trafficking of B cells towards sites of inflammation during a pathogenic exposure. In this regard, previous transcriptional studies have revealed an up-regulation of CK9 in gills in response to a VHSV bath infection [13] and in the intestinal tract after oral vaccination or infection with infectious pancreatic necrosis virus (IPNV), a virus with marked tropism for the digestive system [14]. In contrast, CK9 mRNA levels were not modulated in spleen or head kidney when VHSV or IPNV were injected intraperitoneally [12], but were significantly increased locally after an intraperitoneal virus injection [48]. Although teleost IgT^+^ B cells are present in all immune tissues in physiological conditions, they have been shown to play an important role in mucosal immunity [31] in that the number of IgT^+^ cells in mucosal surfaces strongly increase in response to different pathogenic encounters [31, 32]. On the other hand, IgM^+^ B lymphocytes have also been shown to be recruited to mucosal sites upon antigenic exposure [49]. Furthermore, in contrast to what occurs in mammals, in teleost fish, B lymphocytes, and specially IgM^+^ cells, seem to be one of the main responders to inflammation, being directly mobilized to inflammation sites. For example, IgM^+^ B cells are known to be recruited to the peritoneum after a bacterial injection, representing around two thirds of the recruited cells after 48 h [50] and are one of the main cells that infiltrate the muscle after DNA vaccination [51]. Taken together, it seems that CK9 likely plays a key role in the mobilization of both IgM^+^ and IgT^+^ B lymphocytes from central immune organs to mucosal surfaces or sites of inflammation. The kidney is the main hematopoietic organ in fish and consequently contains a wider variability of leukocyte types, including different myeloid populations such as granulocytes and macrophages [52]. Accordingly, in this organ CK9 also attracts a large subpopulation of MHC II^+^ cells that are not B cells, possibly DCs or macrophages. These cells might also be recruited by CK9 to the periphery, where, together with B cells, they can capture and present specific antigens.

Unlike other mammalian chemokines [40], CK9 showed no lymphoproliferative effects on its own, nor was it capable of modulating the lymphoproliferative effects of other stimuli. Nevertheless, it produced a marked increase of IgT^+^ B lymphocyte survival that was not observed in the case of IgM^+^ B cells. This result indicates that although CK9 is able to attract both B lymphocyte subsets, the activation/differentiation signals it provokes in the cells differ considerably and again points to an important mucosal role for this chemokine. Similarly, CK9 up-regulated the phagocytic capacity of IgM^+^ B cells by increasing the percentage of cells with phagocytic potential, but this effect was not observed in IgT^+^ B cells. Thus, it would be interesting to know if the CK9 receptor signaling in these two cell types is different. In the case that trout CCR9, as expected, is the receptor for CK9, it is interesting to note that two CCR9 molecules with marked differences in their transcriptional regulation have been reported in trout [53]. On the other hand, similar effects of CK9 were observed in both B lymphocyte subsets concerning the regulation of MHC II expression on the cell membrane. This CK9-induced downregulation of MHC II on the cell membrane seemed contradictory at first with the increased phagocytic capacity observed in IgM^+^ cells. Therefore, we decided to measure total MHC II protein levels in parallel, and found that total levels remained unaffected. It is known that in mammals, in contrast to other APCs, resting B cells accumulate most of the MHC II molecules at the cell surface and very little resides in endosomal compartments, whereas, upon activation, MHC II is redistributed to antigen processing compartments [54]. Given our results, it seems that CK9 activates the trafficking of MHC II molecules to endosomal compartments, which should increase antigen loading thus enhancing antigen presenting capacities.

Finally, one of the most interesting results from our study was the fact that BCR cross-linking, induced by incubation with anti-IgM, reduced the migratory capacity of IgM^+^ B lymphocytes. In contrast to the stimulatory effects provoked by a T-independent antigen, BCR engagement significantly reduced the capacity of B cells to migrate towards CK9. As occurs in mammals, BCR cross-linking activates B lymphocytes, inducing intracellular calcium mobilization. Surprisingly, this blocking effect was not observed with anti-IgT, but this lack of response could be due to the lower numbers of IgT^+^ cells in these tissues and/or lower cross-linking potential of the antibody, which resulted in a rather weak intracellular calcium mobilization. This inhibition of B cell migration in response to antigen engagement has also been reported in mammals for chemokines such as CXCL12 [55-57], a consequence of a marked decrease of CXCR4 membrane expression upon stimulation [55-57] which is independent of calcium mobilization, but is mediated by protein kinase C (PKC) [56]. In contrast, the inhibition of CK9 responsiveness by BCR engagement was strongly dependent on calcium mobilization, as incubation with ionomycin completely blocked the migration in the cultures.

In summary, the functional assays performed with CK9 reveal that this chemokine is a strong chemoattractant for both B lymphocytes and macrophages. Consequently, it regulates some of the shared functions between these cells types, such as their phagocytic capacity and their antigen presenting properties (through modulation of MHC II). Taking into account these results, as well as previous transcriptional data that points to a preferential induction of CK9 in mucosal sites, this chemokine is likely to play an important role in the mobilization of both IgM^+^ and IgT^+^ B cell subpopulations to mucosal sites, and the survival of IgT^+^ B lymphocytes at these sites. This capacity of B cells to migrate to CK9 was increased by a T-independent antigen exposure but was repressed by BCR-engagement, suggesting that once a B cell has found a high affinity cognate antigen, CK9 unresponsiveness traps these cells within the mucosa to start a local immune response.

## MATERIALS AND METHODS

### Experimental fish

Female rainbow trout (*Oncorhynchus mykiss*) adults of ~100 g were obtained from Centro de Acuicultura El Molino (Madrid, Spain) and maintained at the animal facilities of the Centro de Investigación en Sanidad Animal in a recirculating water system at 16°C, with 12:12 h light/dark photoperiod. Fish were fed twice a day with a commercial diet (Skretting). Prior to any experimental procedure, fish were acclimatized to laboratory conditions for at least 2 weeks. All of the experiments described comply with the Guidelines of the European Union Council (2010/63/EU) for the use of laboratory animals and have been approved by the Instituto Nacional de Investigación Agraria y Alimentaria (INIA) Ethics Committee.

### Production of recombinant CK9

The coding region of the predicted mature CK9 peptide (Acc. Nos. BX316999 and BX855929) was amplified from a plasmid clone using primers (forward: CAAGGCTCTTATGGGAACTGCTGT and reverse: GTTGCTTTGCTGCTTATCAACAACAGT) and the Q5 high fidelity enzyme (New England Biolabs, UK). The amplified product was cloned to a pET vector (Invitrogen). The construct (pET-CK9) encodes an identical CK9 mature amino acid sequence with the addition of methionine at the N-terminus and a his-tag (GSGHHHHHHHH) added at the C-terminus for purification. Thus, the recombinant trout CK9 is 84 aa, with a calculated molecular weight of 9.61 kDa and a theoretical pI of 9.79. Following transformation of the pET-CK9 plasmid into BL21 Star (DE3) competent cells (Invitrogen), the induction of recombinant protein production, purification under denaturing conditions, refolding, re-purification under native conditions, SDS-PAGE analysis of proteins and quantification of protein concentration were as described previously [35, 58, 59]. The refolding buffer contained 50 mM Tris-HCl (pH7.5), 10% glycerol, 0.6 M arginine monohydrochloride, 1 M 3-(1-Pyridinio)-1-propanesulfonate (known as NDSB 201 and PPS, Sigma), 0.2% PEG3350 and 5 mM 2-mercaptoethanol. The purified protein was desalted in desalting buffer (DSB) (PBS containing 50% glycerol) using PD-10 Desalting Columns (GE Healthcare). After sterilization with a 0.2 μm filter, the rCK9 (0.6 mg/ml) was aliquoted and stored at −80°C ready for stimulation of cells.

### Leukocyte isolation and cell lines

Rainbow trout were killed by MS-222 (Sigma) overdose and blood was extracted with a heparinized needle from the caudal vein and diluted 10 times with L-15 medium (Invitrogen) supplemented with 100 I.U./ml penicillin plus 100 μg/ml streptomycin (P/S, Life Technologies), 10 units/ml heparin (Sigma) and 5% FCS (Life Technologies). Single cell suspensions from spleen and head kidney were obtained using 100 μm nylon cell strainers (BD Biosciences), placed onto 30/51% discontinuous Percoll (GE Healthcare) density gradients and centrifuged at 500 x *g* for 30 min at 4°C. The cells at the interface were collected and washed twice in L-15 containing 5% FCS.

RTS11, a continuous rainbow trout macrophage-like cell line, originally isolated from a long-term spleen hematopoietic culture [60] was maintained at 18°C in L-15 medium supplemented with P/S and 15% FCS. Cells were grown at a high cell density and passaged at a 1:2 ratio as described previously [60].

### Flow cytometry

The anti-trout CD8α (mAb rat IgG, 7 μg/ml), the anti-trout MHC II β-chain [mAb mouse IgG1; used as an Allophycocyanin (APC) fluorescently-labelled version], the anti-trout IgM [1.14, mAb mouse IgG1; used as a Phycoerythrin (PE) fluorescently-labelled version] and the anti-trout IgT (mAb mouse IgG2a; used as a PE fluorescently-labelled version) used in this study have been previously characterized [31, 38, 61, 62]. Primary antibodies were conjugated by using Lightning-Link APC or Lightning-Link R-PE (Innova Biosciences). A mix of corresponding isotype control antibodies (BioLegend) was used in each experiment to rule out non-specific binding. For extracellular staining, isolated cells were incubated for 30 min with primary antibodies, washed twice with staining buffer (PBS containing 1% FCS and 0.5% sodium azide) and stained for 20 min with secondary Ab for anti-CD8α detection [R-PE F(ab')2 fragment of goat anti-rat IgG (H+L) (Life Technologies)] when needed. To determine total (intracellular and extracellular) MHC II levels, cells were fixed for 5 min with 4% paraformaldehyde in PBS, then permeabilized for 30 min in permeabilizitation buffer (staining buffer containing 0.1% saponin). Then, the cells were incubated with MHC II-APC antibody in permeabilization buffer for another 30 min. After incubation, cells were washed three times with staining buffer, and analyzed on a FACSCalibur flow cytometer (BD Biosciences) equipped with CellQuest Pro software. Flow cytometry analysis was performed with FlowJo 10 (TreeStar).

### Chemotaxis assays

Chemotaxis was assessed using 3 μm (for primary cultures) or 5 μm (for RTS11 cells) pore polycarbonate Transwell chambers (Sigma), following the manufacturer's instructions. Briefly, 600 μl of control medium (L-15 medium supplemented with P/S and 5% FCS) or medium containing 1, 10 or 100 ng/ml recombinant CK9 were added to the bottom chambers. In the upper chambers, 100 μl of RTS11 cells or primary cells (2 × 10^6^ cells/ml) were dispensed. To test the specificity of the migration, in some experiments leukocytes were previously incubated with PTX (200 or 500 ng/ml) for 2 h, prior to conducting the chemotaxis assays. When needed, spleen or blood leukocytes were activated with TNP-LPS (5 μg/ml) or TNP-KLH (Keyhole Limpet Hemocyanin) (5 μg/ml) (Sigma) for 24 h at 20°C before conducting the chemotaxis assays. In other experiments, primary cells were pre-stimulated with 0.5 mg/ml of anti-IgM antibody, 0.5 mg/ml of anti-IgT antibody or a combination of both, for 24 h at 14°C, or with 0.5 mg/ml of anti-IgM or 1 mM ionomycin for 5 min at 20°C. Cells in migration chambers were incubated for 2 h at 20°C, then the 600 μl of the bottom chamber were harvested and the migrated cells were analyzed based on side and forward light scatter (SSC/FSC) parameters on a FACSCalibur flow cytometer equipped with CellQuest sofware (BD Biosciences) at a constant flow time (1 min). In some experiments, the migrated cells were stained with mAbs against IgM, IgT, CD8α and MHC II or isotype control when needed and analyzed by flow cytometry.

### Respiratory burst activity

To analyze the respiratory burst activity of RTS11 trout macrophages, we used the Nitro Blue Tetrazolium (NBT, Sigma) method. For this, cells were seeded in 96-well plates (Nunc) at a concentration of 2 × 10^5^ cells per well, washed and resuspended in 1X Phenol Red-Free Hank's Balanced Salt Solution (HBSS, Life Technologies). Then, NBT was added to the cells at a final concentration of 1 μg/ml, in the presence or absence of 100 ng/ml CK9. As a positive inducer of the respiratory burst, 1 μg/ml of PMA (Sigma) was included in some wells. To assess the specificity of the reaction, 300 U/ml of Superoxide Dismutase (SOD, Sigma) was used in some wells in combination with either CK9 or PMA. Cells were incubated for 1 h at room temperature, in the dark, and then centrifuged. The supernatant was discarded and cells were incubated with absolute methanol for 5 min at room temperature. Methanol was then discarded and the cells were air-dried for 30 min. The reaction was developed by the addition of a developing buffer (120 μl of 2 M KOH and 140 μl of DMSO per well). Samples were incubated for 5 min and optical density at 620 nm measured, to determine reduction of the NBT substrate.

### Phagocytic activity

For the analysis of phagocytosis, leukocytes or RTS11 cells were seeded in 96-well plates (Nunc) at a cell density of 2×10^5^ cells per well and incubated for 3 h at 20°C with fluorescent beads (FluoSpheres^®^ Microspheres, 1.0 μm, Crimson Red Fluorescent 625/645, 2% solids; Life Technologies) at a cell:bead ratio of 1:10 in the presence or absence of 100 ng/ml CK9. Cells were harvested using a standard cell scraper (Corning). Non-ingested beads were removed by centrifugation (100 x *g* for 10 min at 4°C) over a cushion of 3% (weight/volume) BSA (Fraction V; Fisher Scientific) in PBS supplemented with 4.5% (weight/volume) D-glucose (Sigma). Cells were resuspended in staining buffer (PBS containing 1% FCS and 0.5% sodium azide), labelled with the flow cytometry antibodies when needed, and analyzed on a FACSCalibur flow cytometer.

### Cell proliferation

A BrdU Flow kit (BD Biosciences) was used to measure the specific proliferation of IgM^+^ and IgT^+^ B cells, following the manufacturer's instructions. Spleen or blood leukocytes at a concentration of 2 × 10^6^ cells per ml were incubated for 3 days at 20°C in control medium or medium containing the different stimuli and/or CK9 (100 ng/ml). As lymphoproliferative stimuli, TNP-LPS (5 μg/ml) or TNP-KLH (5 μg/ml) were used. After this time, bromodeoxyuridine (BrdU) (10 μM) was added to the cultures, and cells were incubated for an additional 24 h. Trout cells were collected and stained with anti-IgM-PE or anti-IgT-PE MAbs and then fixed and permeabilized following the manufacturer's instructions. In brief, cells were fixed with Cytofix/Cytoperm buffer for 15 min on ice, incubated with Cytoperm Permeabilization Buffer Plus for 10 min on ice and refixed with Cytofix/Cytoperm buffer during 5 min at room temperature. Cells were then incubated with DNase (30 μg/10^6^ cells) for 1 h at 37°C to expose the incorporated BrdU. Finally, the cells were stained with FITC anti-BrdU mAb and analyzed by flow cytometry.

### Calcium flux analysis

For calcium flux analysis, the calcium indicator Fluo-3 AM (Life Technologies) was used, following the manufacturer's instructions. Briefly, Fluo-3 was dissolved in DMSO and further diluted in an equal volume of 20% (w/v) Pluronic F-127 (Life Technologies). Cells were incubated with Fluo-3 AM at a final concentration of 5 μM, diluted in L-15 medium without FCS, for 60 min. After collecting and washing the cells with staining buffer, a baseline reading for 45 s was acquired by flow cytometry and then 0.5 μg/ml of anti-IgM, 0.5 μg/ml of anti-IgT or 1 μM ionomycin added to the sample tube. The emission of fluorescence (525 nm) was then immediately recorded for 300 s.

### Statistical analysis

Data handling, analyses and graphic representation was performed using Microsoft Office Excel 2010. Statistical analyses were performed using two tailed unpaired Student's *t* tests, after having confirmed that populations did not display statistically different variances. The differences between the mean values were considered significant when *P* < 0.05, where * means *P* < 0.05, ** means P < 0.01 and *** means P < 0.005.

## SUPPLEMENTARY MATERIAL FIGURE



## Abbreviations

APCs: antigen presenting cells
BCR: B cell receptor
DCs: dendritic cells
IL: interleukin
LPS: lipopolysaccharide
MIP: macrophage inflammatory protein
MHC II: major histocompatibility complex class II
MCP: monocyte chemotactic protein
TLR: Toll-like receptor
TNF-α: tumor necrosis factor α
