# Supplementary material for: Rainbow trout CK9, a CCL25-like ancient chemokine that attracts and regulates B cells and macrophages, the main antigen presenting cells in fish
Source: Oncotarget. 2016 Mar 17;7(14):17547–64. doi: 10.18632/oncotarget.8163 (PMC4951232; doi:10.18632/oncotarget.8163)
Supplement: Supplementary file 1 [file oncotarget-07-17547-s001.pdf]

Rainbow trout CK9, a CCL25-like ancient chemokine that attracts and regulates B cells and macrophages, the main antigen presenting cells in fish

Supplementary Material

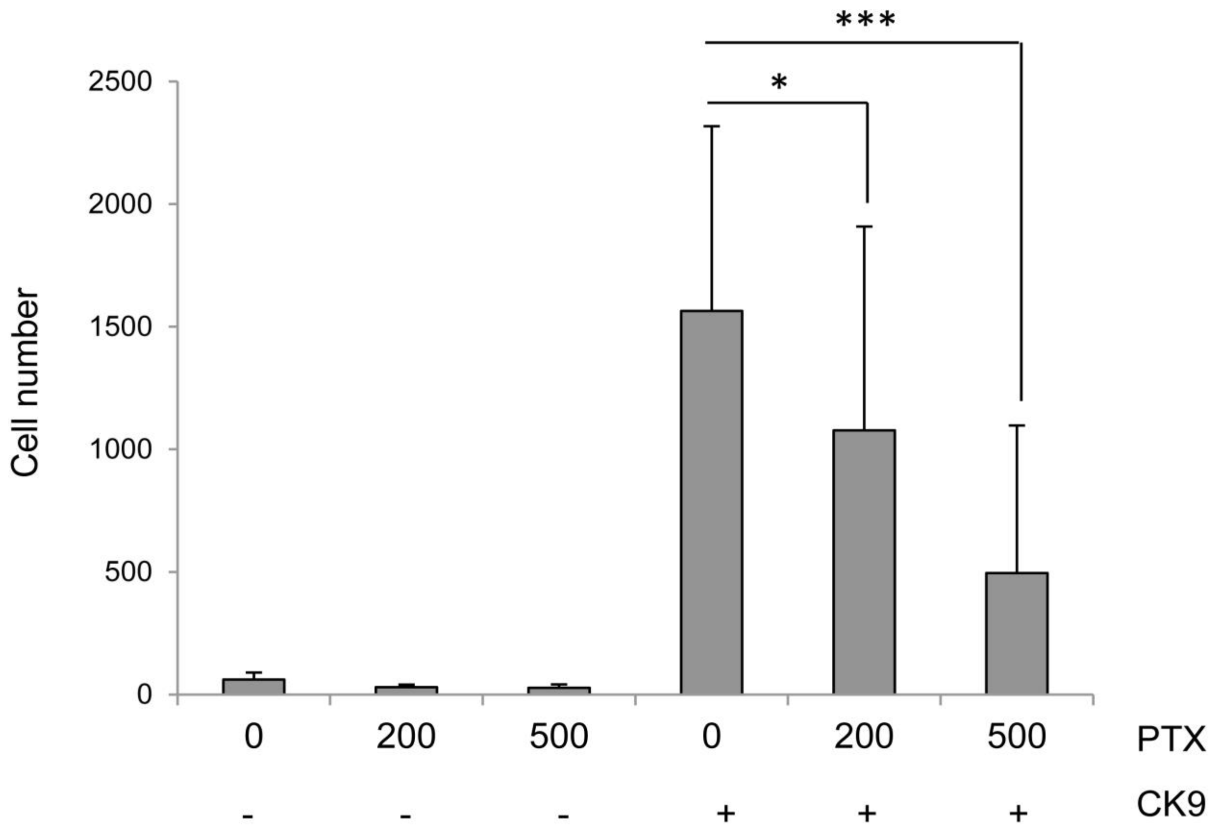

Fig. S1. Chemotactic activity of CK9 is blocked by pertussis toxin (PTX). Splenocytes were pre-incubated for 2 h at 20ºC with 200 or 500 ng/ml of PTX or left untreated. After that time, the cells were introduced into the upper wells of transwell chambers, whereas 100 ng/ml CK9 or media alone were introduced into the bottom wells of the chambers. After 2 h of incubation at 20ºC, the number of cells that had migrated to the bottom of the wells was quantified by flow cytometry. Average numbers of migrating cells (n=5 fish, mean + SD) are shown. Statistical analysis was performed in each case, where * means $p \leq 0.05$ and *** means $p \leq 0.005$**.**
